# Supplementary material for: Physics-Based Simulations to Predict the Differential Effects of Motor Control and Musculoskeletal Deficits on Gait Dysfunction in Cerebral Palsy: A Retrospective Case Study
Source: Front Hum Neurosci. 2020 Feb 18;14:40. doi: 10.3389/fnhum.2020.00040 (PMC7040166; doi:10.3389/fnhum.2020.00040)
Supplement: Supplementary file 1 [file Table_1.PDF]

Supplementary Tables

Table S1. Generic and personalized optimal fiber lengths (cm). L and R are for left and right, respectively. Lh and sh are for long and short head, respectively.

	**Gluteus maximus 1**		**Gluteus maximus 2**		**Gluteus maximus 3**		**Gluteus medius 1**		**Gluteus medius 2**	
	L	R	L	R	L	R	L	R	L	R
**Generic**	13.35	13.44	12.93	12.95	12.85	12.72	6.23	6.11	10.36	10.34
**Personalized**	6.67	6.72	6.47	6.47	7.99	7.61	3.11	3.05	5.18	5.45
	**Gluteus medius 3**		**Gluteus minimus 1**		**Gluteus minimus 2**		**Gluteus minimus 3**		**Adductor longus**	
	L	R	L	R	L	R	L	R	L	R
**Generic**	6.82	6.76	6.80	6.99	6.81	6.93	3.66	3.62	13.01	12.40
**Personalized**	3.55	3.38	4.56	4.80	3.73	3.93	2.05	1.95	6.51	6.20
	**Adductor brevis**		**Adductor magnus 1**		**Adductor magnus 2**		**Adductor magnus 3**		**Pectineus**	
	L	R	L	R	L	R	L	R	L	R
**Generic**	13.93	13.08	9.10	8.29	11.67	11.19	12.63	13.13	16.06	14.41
**Personalized**	8.68	8.27	7.55	7.19	5.88	5.60	6.63	6.56	10.04	9.57
	**Iliacus**		**Psoas**		**Quadratus femoris**		**Gemellus**		**Piriformis**	
	L	R	L	R	L	R	L	R	L	R
**Generic**	10.35	10.06	9.50	9.35	3.71	3.72	1.67	1.57	2.28	2.31
**Personalized**	5.17	5.03	4.75	4.68	4.16	3.96	1.90	1.81	1.21	1.15
	**Tensor fasciae latae**		**Gracilis**		**Semimembranosus**		**Semitendinosus**		**Biceps femoris lh**	
	L	R	L	R	L	R	L	R	L	R
**Generic**	8.81	8.88	33.36	33.23	7.48	7.50	18.32	18.42	10.05	10.08
**Personalized**	9.28	8.84	16.68	16.61	7.88	7.75	10.26	10.80	10.03	10.48
	**Biceps femoris sh**		**Sartorius**		**Rectus femoris**		**Vastus medius**		**Vastus intermedius**	
	L	R	L	R	L	R	L	R	L	R
**Generic**	13.54	14.08	56.42	56.73	7.74	7.73	8.09	8.02	7.66	7.59
**Personalized**	7.39	7.04	38.74	36.89	8.16	8.59	4.93	4.70	4.76	4.73
	**Vastus lateralis**		**Gastrocnemius medialis**		**Gastrocnemius lateralis**		**Soleus**		**Tibialis posterior**	
	L	R	L	R	L	R	L	R	L	R
**Generic**	7.49	7.43	3.86	3.89	5.56	5.58	2.62	2.64	2.72	2.74
**Personalized**	6.50	6.23	3.26	3.43	3.21	3.38	1.93	2.03	1.44	1.37
	**Tibialis anterior**		**Extensor digitalis**		**Extensor hallucis**		**Flexor digitalis**		**Flexor hallucis**	
	L	R	L	R	L	R	L	R	L	R
**Generic**	8.45	8.52	8.83	8.84	9.51	9.54	2.90	2.91	3.64	3.65
**Personalized**	4.22	4.26	4.64	4.42	5.01	4.77	1.53	1.45	1.92	1.83
	**Peroneus brevis**		**Peroneus longus**		**Peroneus tertius**					
	L	R	L	R	L	R				
**Generic**	4.15	4.23	4.26	4.28	6.32	6.50				
**Personalized**	2.19	2.12	2.24	2.14	3.35	3.25				

Table S2. Generic and personalized tendon slack lengths (cm). L and R are for left and right, respectively. Lh and sh are for long and short head, respectively.

	**Gluteus maximus 1**		**Gluteus maximus 2**		**Gluteus maximus 3**		**Gluteus medius 1**		**Gluteus medius 2**	
	L	R	L	R	L	R	L	R	L	R
**Generic**	11.75	11.83	11.17	11.18	10.18	10.07	9.08	8.90	4.54	4.53
**Personalized**	11.34	11.93	12.01	11.92	13.23	13.51	9.94	10.46	8.35	8.79
	**Gluteus medius 3**		**Gluteus minimus 1**		**Gluteus minimus 2**		**Gluteus minimus 3**		**Adductor longus**	
	L	R	L	R	L	R	L	R	L	R
**Generic**	5.60	5.55	1.6	1.64	3.16	3.22	4.92	4.85	10.42	9.93
**Personalized**	8.47	8.48	3.12	3.29	6.11	6.43	7.06	6.88	11.85	11.29
	**Adductor brevis**		**Adductor magnus 1**		**Adductor magnus 2**		**Adductor magnus 3**		**Pectineus**	
	L	R	L	R	L	R	L	R	L	R
**Generic**	1.36	1.28	6.27	5.72	12.54	12.03	25.07	26.06	0.12	0.11
**Personalized**	2.69	2.56	4.81	4.58	13.12	12.50	27.07	25.78	0.23	0.22
	**Iliacus**		**Psoas**		**Quadratus femoris**		**Gemellus**		**Piriformis**	
	L	R	L	R	L	R	L	R	L	R
**Generic**	9.31	9.05	10.33	10.16	1.65	1.66	2.71	2.55	9.29	9.40
**Personalized**	13.49	13.39	15.19	14.93	1.37	1.30	3.47	3.30	10.40	10.85
	**Tensor fasciae latae**		**Gracilis**		**Semimembranosus**		**Semitendinosus**		**Biceps femoris lh**	
	L	R	L	R	L	R	L	R	L	R
**Generic**	39.41	39.73	13.27	13.22	33.56	33.64	23.88	24.01	31.45	31.53
**Personalized**	37.94	36.14	20.25	20.21	30.10	31.68	30.11	31.69	28.75	30.26
	**Biceps femoris sh**		**Sartorius**		**Rectus femoris**		**Vastus medius**		**Vastus intermedius**	
	L	R	L	R	L	R	L	R	L	R
**Generic**	7.83	8.14	3.90	3.92	31.88	31.85	11.46	11.36	11.97	11.87
**Personalized**	10.38	10.93	7.80	7.42	28.93	29.40	14.25	14.95	14.30	15.05
	**Vastus lateralis**		**Gastrocnemius medialis**		**Gastrocnemius lateralis**		**Soleus**		**Tibialis posterior**	
	L	R	L	R	L	R	L	R	L	R
**Generic**	14.01	13.88	34.99	35.23	33.44	33.56	23.41	23.61	27.22	27.35
**Personalized**	14.13	14.87	33.23	34.59	33.38	34.48	23.53	23.52	29.27	29.16
	**Tibialis anterior**		**Extensor digitalis**		**Extensor hallucis**		**Flexor digitalis**		**Flexor hallucis**	
	L	R	L	R	L	R	L	R	L	R
**Generic**	19.23	19.39	29.86	29.91	26.12	26.21	34.07	34.18	32.18	32.3
**Personalized**	20.40	21.48	30.93	31.75	27.67	28.43	35.60	35.49	34.12	33.87
	**Peroneus brevis**		**Peroneus longus**		**Peroneus tertius**					
	L	R	L	R	L	R				
**Generic**	13.37	13.63	30.01	30.11	8.00	8.23				
**Personalized**	15.09	15.88	31.25	31.40	9.66	10.17				
