# Supplementary material for: Physics-Based Simulations to Predict the Differential Effects of Motor Control and Musculoskeletal Deficits on Gait Dysfunction in Cerebral Palsy: A Retrospective Case Study
Source: Front Hum Neurosci. 2020 Feb 18;14:40. doi: 10.3389/fnhum.2020.00040 (PMC7040166; doi:10.3389/fnhum.2020.00040)
Supplement: Supplementary file 10 [file Image_1.PDF]

frontiers

Supplementary Figures

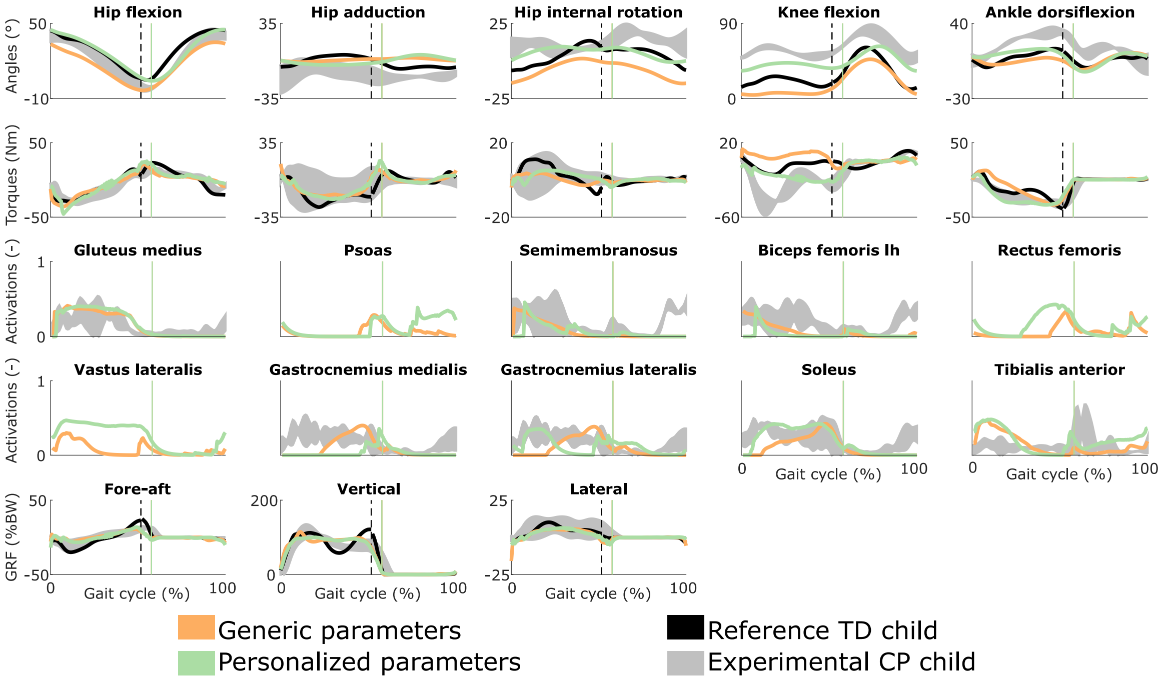

Figure S1. Influence of the muscle-tendon parameters on the predicted walking gaits. Variables from the left leg are shown over a complete gait cycle; right leg variables are shown in Figure 2 (Manuscript). Solid vertical lines indicate the transition from stance to swing. Experimental data is shown as mean ± two standard deviations. Experimental EMG data was normalized to peak activations. Reference TD child data was available for a single gait cycle starting at right heel strike; left leg data was thus reconstructed from that gait cycle but is discontinuous as indicated by the dashed vertical lines. GRF is for ground reaction forces; BW is for body weight; COT is for metabolic cost of transport; lh is for long head.

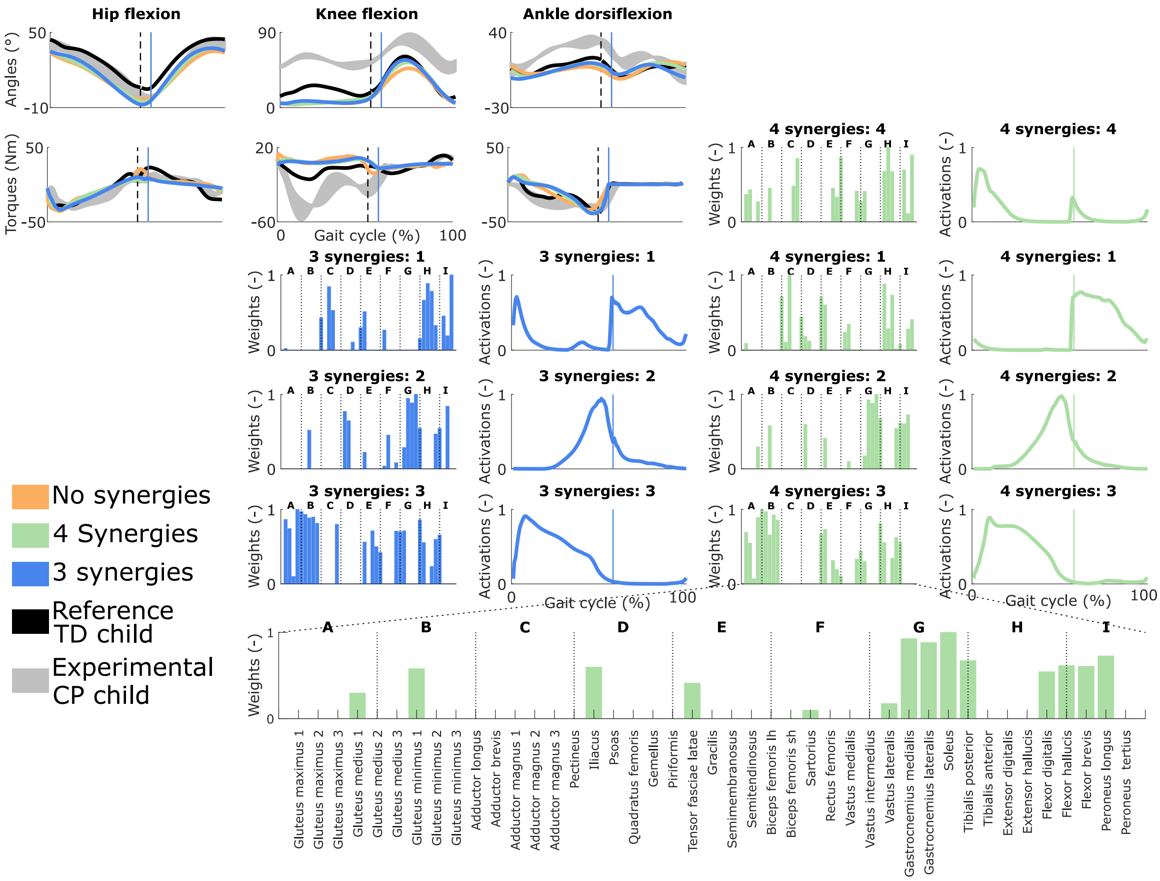

Figure S2. Influence of the synergies on walking gaits predicted with the generic muscle-tendon parameters. Variables from the left leg are shown over a complete gait cycle; right leg variables are shown in Figure 3 (Manuscript). Vertical lines (solid) indicate the transition from stance to swing. Panels of synergy weights are divided into sections (A-I) to relate bars to muscle names provided in the bottom bar plot, which is an expanded version of the plot of weights with title 4 synergies: 3. Lh and sh are for long and short head, respectively. Weights were normalized to one. Experimental data is shown as mean ± two standard deviations. Reference TD child data was available for a single gait cycle starting at right heel strike; left leg data was thus reconstructed from that gait cycle but is discontinuous as indicated by the dashed vertical lines.

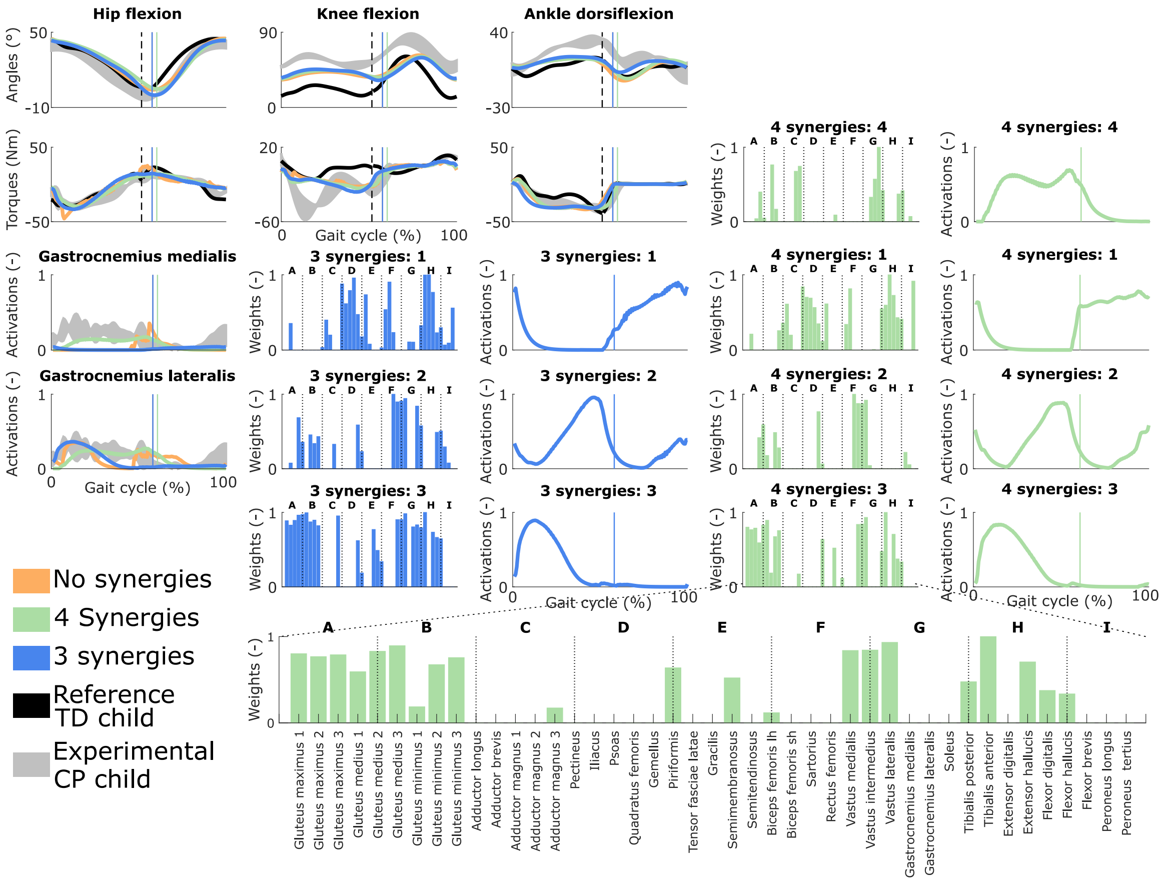

Figure S3. Influence of the synergies on walking gaits predicted with the personalized muscle-tendon parameters. Variables from the left leg are shown over a complete gait cycle; right leg variables are shown in Figure 4 (Manuscript). Vertical lines (solid) indicate the transition from stance to swing. Panels of synergy weights are divided into sections (A-I) to relate bars to muscle names provided in the bottom bar plot, which is an expanded version of the plot of weights with title 4 synergies: 3. Lh and sh are for long and short head, respectively. Weights were normalized to one. Experimental data is shown as mean ± two standard deviations. Experimental EMG data was normalized to peak activations. Reference TD child data was available for a single gait cycle starting at right heel strike; left leg data was thus reconstructed from that gait cycle but is discontinuous as indicated by the dashed vertical lines.

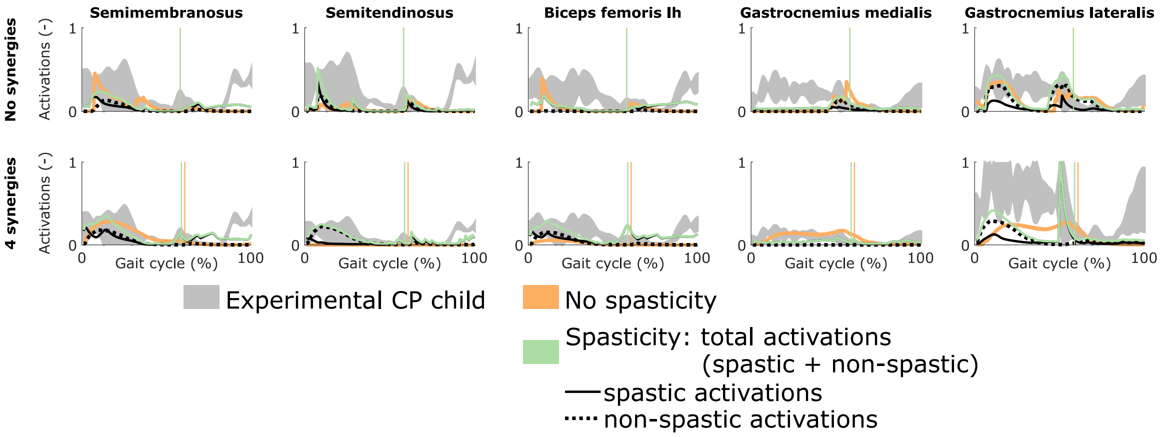

Figure S4. Influence of spasticity on the predicted muscle activity. Activations from left leg muscles only are shown over a complete gait cycle; right leg activations are shown in Figure 5 (Manuscript). When accounting for spasticity, total activations (green) combine spastic (solid black) and non-spastic (dotted black) activations. Vertical lines indicate the transition from stance to swing. Experimental data is shown as mean ± two standard deviations. Experimental EMG data was normalized to peak activations. Lh is for long head.

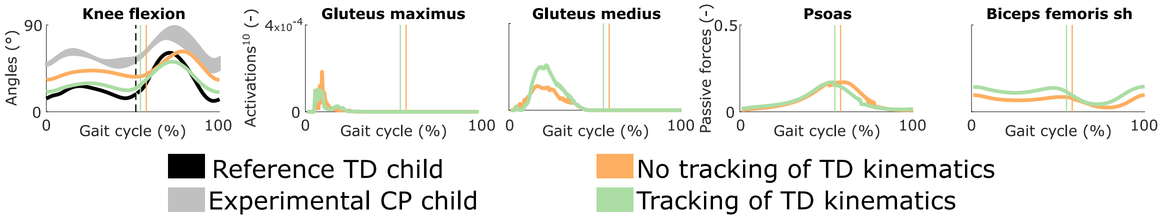

Figure S5. Influence of tracking the TD kinematics on predicted walking gaits. Variables from the left leg are shown over a complete gait cycle; right leg variables are shown in Figure 6 (Manuscript). Vertical lines indicate the transition from stance to swing. Experimental data is shown as mean ± two standard deviations. Muscle fatigue is modeled by activations at the tenth power. Passive muscle forces are normalized by maximal isometric muscle forces. Sh is for short head.

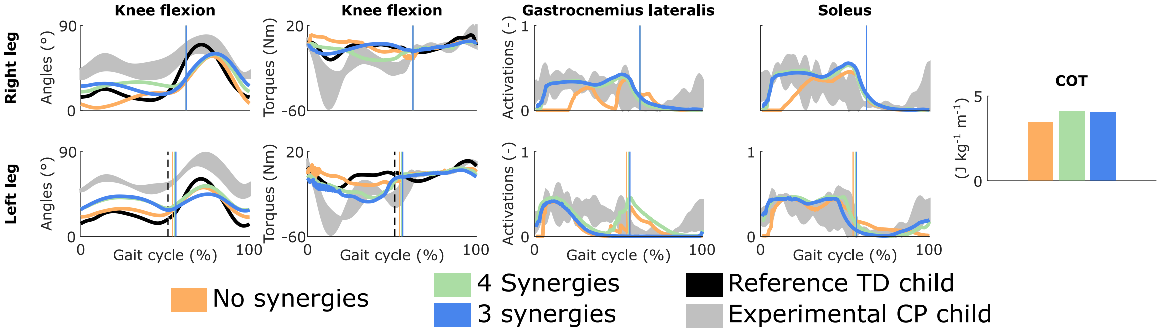

Figure S6. Influence of the synergies on walking gaits predicted with the personalized muscle-tendon parameters while tracking the TD kinematics. Solid vertical lines indicate the transition from stance to swing. Experimental data is shown as mean ± two standard deviations. Experimental EMG data was normalized to peak activations. Reference TD child data was available for a single gait cycle starting at right heel strike; left leg data was thus reconstructed from that gait cycle but is discontinuous as indicated by the dashed vertical lines.
